# Inhibition of Multidrug Resistance by SV40 Pseudovirion Delivery of an Antigene Peptide Nucleic Acid (PNA) in Cultured Cells

**DOI:** 10.1371/journal.pone.0017981

**Published:** 2011-03-22

**Authors:** Benjamin Macadangdang, Ning Zhang, Paul E. Lund, Andrew H. Marple, Mitsunori Okabe, Michael M. Gottesman, Daniel H. Appella, Chava Kimchi-Sarfaty

**Affiliations:** 1 Laboratory of Cell Biology, National Cancer Institute, National Institutes of Health, Bethesda, Maryland, United States of America; 2 Laboratory of Bioorganic Chemistry, National Institute of Diabetes and Digestive and Kidney Diseases, National Institutes of Health, U.S. Department of Health & Human Services, Bethesda, Maryland, United States of America; 3 Center for Biologics Evaluation and Research, Food and Drug Administration, Bethesda, Maryland, United States of America; Deutsches Krebsforschungszentrum, Germany

## Abstract

Peptide nucleic acid (PNA) is known to bind with extraordinarily high affinity and sequence-specificity to complementary nucleic acid sequences and can be used to suppress gene expression. However, effective delivery into cells is a major obstacle to the development of PNA for gene therapy applications. Here, we present a novel method for the *in vitro* delivery of antigene PNA to cells. By using a nucleocapsid protein derived from Simian virus 40, we have been able to package PNA into pseudovirions, facilitating the delivery of the packaged PNA into cells. We demonstrate that this system can be used effectively to suppress gene expression associated with multidrug resistance in cancer cells, as shown by RT-PCR, flow cytometry, Western blotting, and cell viability under chemotherapy. The combination of PNA with the SV40-based delivery system is a method for suppressing a gene of interest that could be broadly applied to numerous targets.

## Introduction

The effectiveness of chemotherapeutic agents is often limited by various mechanisms that exist in cancer cells, often through overexpression of certain genes, in particular those coding for membrane-spanning ATP-binding cassette (ABC) transporters; examples include *MDR*1 [1], ABCG2 [2]–[4], and *MRP1* [5]. The *MDR*1 gene is frequently overexpressed in several drug-resistant cancers, such as acute myeloid leukemia [6], [7], colon cancer [8], adrenal cancer [9], and kidney cancer [10]. An over-abundance of P-glycoprotein (P-gp), the protein product of the *MDR*1 gene [1], leads to multidrug resistance (MDR), because cancer cells become able to efflux a number of structurally diverse compounds including many chemotherapy agents, such as paclitaxel [11], [12], doxorubicin [13], and vinblastine [14]. Silencing the expression of genes such as *MDR*1 is one potential way to address the problem of MDR in cancer.

Peptide nucleic acid (PNA) is a synthetic DNA analog in which the sugar-phosphate backbone is replaced with a polyamide backbone [15], [16]. The nucleobases covalently attached to the PNA backbone enable the PNA oligomers to bind to complementary DNA or RNA sequences via Watson-Crick base-pairing. Certain key properties of PNA make it ideal to target DNA or RNA sequences *in vivo* [17]. First, because PNA is purely synthetic, it is resistant to degradation by nucleases and proteases [18], and thus these molecules may remain in cells for extended time periods [19]. Second, the thermal stability of PNA∶oligonucleotide complexes is significantly higher than corresponding oligonucleotide duplexes. This high stability when bound to its target oligonucleotide should enhance the ability of PNA to suppress protein or gene expression. Numerous studies have sought to develop PNA as an antisense agent to suppress protein expression by targeting an mRNA sequence [20]. In this way, PNA inhibits translation by sterically blocking translation start sites along mRNA [21]. More recently, PNA has been used as an antigene agent that suppresses gene expression by targeting a DNA sequence [22]. The work of the Corey group [23] has elegantly demonstrated that PNA designed to target the transcription initiation sites of genes may effectively suppress overall expression; by targeting the transcription start site of the gene, the whole gene is inhibited, including all splicing forms of the protein, making antigene PNA a powerful inhibitor.

To date, only a few genes have been targeted using this approach. A key hurdle in the development of PNA as an antisense or antigene agent is the effective delivery of PNA to cells [24]. While some cellular systems are permeable to PNA, many cells lines are not [15], [17], [21], so various systems have been developed to deliver PNA to cells. Most of these systems involve covalent conjugation of PNA to another molecule that facilitates delivery into a cell, such as a lipid or cell-penetrating peptide [22], [25]–[30]. Others chemically modify the PNA backbone with multiple arginine side chains to gain entry [31]. Unfortunately, there is no delivery system currently available that works with all cells. Therefore, a universally applicable system could facilitate the therapeutic use of PNA.

To that end, we investigated the simian virus 40 (SV40) *in vitro*-packaging (IVP) system, in which pseudovirions are formed when the major capsid protein of SV40 (VP1) self-assembles around nucleic acids [32], [33]. Unlike other *in-vitro* packaging systems, no viral genetic material or packaging signal sequence is required to form these pseudovirions. To date, SV40 pseudovirions have been shown to deliver reporter genes such as GFP, suicide genes such as *Pseudomonas exotoxin*, plasmids encoding shRNA, and siRNA oligomers [34], [35]. These pseudovirions can transduce both dividing and quiescent cells *in vitro* and tissues *in vivo*. Therefore, we chose the SV40 system to deliver PNA molecules to cancer cells.

Here we demonstrate the effectiveness of the SV40 IVP system to deliver antigene PNA molecules against the *MDR*1 gene through the reduction of the expression of *MDR*1 at the transcriptional level leading to reduced total levels of P-gp within a cell. These changes result in a decreased ability of the cell to efflux xenobiotic compounds (efflux capacity). Additionally, drug-resistant cells transduced with PNA targeted against the *MDR*1 gene and treated with the chemotherapeutic agent paclitaxel were significantly less viable compared to those transduced with a scrambled sequence of PNA. Our *in vitro* results suggest that delivery of PNA via the SV40 delivery system would be a promising technique to treat certain cancers that exhibit *MDR*1-mediated MDR.

## Results and Discussion

### Antigene PNA silences MDR1 mRNA transcription

A scrambled PNA sequence (**S**), and an antigene PNA (**P**) designed to bind to a sequence in the vicinity (−2 to +13) of the major transcription start site of *MDR*1 were designed. The stated position of **P** is relative to the major +1 transcription start site (nt 87,230,200 of the NCBI Reference Sequence NC_000007.13) of the *MDR1* gene as described by Ueda *et al.* [36]. A detailed BLAST summary of the two sequences indicates that **P** maps only to the *MDR*1 gene with 100% coverage while **S** does not map to any coding transcript with 100% coverage. After synthesis and purification by reverse-phase high performance liquid chromatography, the identity of the PNAs was confirmed by mass spectroscopy and the melting temperatures of complementary PNA∶DNA duplexes were determined (Table 1). The KB-8-5 cell line was chosen for subsequent analysis because it is a human KB carcincoma cell line that is known to overexpress P-gp and is thus resistant to a variety of chemotherapeutic agents.

**Table 1:** PNA Sequences, Melting Temperatures, and Mass Spectroscopy Characterization.

PNA	Sequence	*T_m_* a (°C)	Molecular Weight^b^	
			Calculated	Observed
**P**	AcNH-TCA TTC GAG TAG CGG-Lys-CONH_2_	76.5	4288.1	4288.3
**S**	AcNH-TAC GTC ATC TCG CAG-Lys-CONH_2_	72.0	4208.1	4207.5

Ac = acetyl group.

a *T*_m_ represents the melting temperature for the duplex formed between the indicated PNA and antiparallel DNA. Conditions for *T*_m_ measurement were as follows: 3.0 µM of PNA∶DNA duplex, 150 mM NaCl, 10 mM sodium phosphate buffer, pH 7.0, 0.1 mM EDTA, UV measured at 260 nm from 95 to 25°C, in 1°C increments. All values are averages from two or more experiments.

b Accurate mass Electrospray Ionization (ESI) mass spectra were obtained on a Waters LCT Premier time-of-flight (TOF) mass spectrometer. The instrument was operated in W-mode at a nominal resolution of 10000. The electrospray capillary voltage was 2 KV and the sample cone voltage was 60 volts. The desolvation temperature was 275°C and the desolvation gas was nitrogen with a flow rate of 300 L/hr. Accurate masses were obtained using a standard internal reference. The sample was introduced into the mass spectrometer via a direct loop injection. Both positive and negative ion accurate mass data were achieved simply by reversing the instrument's operating polarity. Deconvolution of multiply charged ions was performed with MaxEnt I.

**Figure 1:**
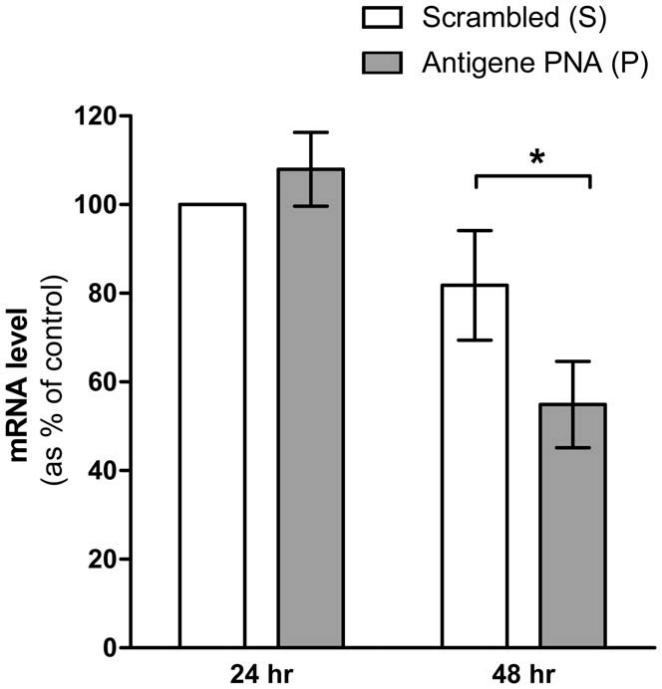
*MDR*1 mRNA levels in KB-8-5 cells 24 hr and 48 hr post transduction with IVP-scrambled (S) or IVP-antigene PNA (P). *MDR*1 mRNA levels were normalized by *GAPDH* levels, and are reported relative to the *MDR*1 mRNA level of cells transduced with **S**, 24 hr post transduction. A significant difference (*, p<0.05) was observed between **P** and **S** at 48 hr post transduction. Error bars represent the standard deviation from the average of three experiments.

We first sought to measure how efficient the antisense PNA molecules were at blocking the transcription of the *MDR*1 gene by measuring the *MDR*1 mRNA levels in KB-8-5 cells transduced with **P** and **S** via the SV40 delivery system. The mRNA levels were quantified using RT-PCR 24 hr and 48 hr post transduction and normalized to the housekeeping gene *GAPDH*. A slight increase was observed after 24 hr (p>0.1), but a significant reduction (33%, p<0.05) in *MDR*1 mRNA levels was observed 48 hr post-transduction in cells transduced with **P** as compared to those transduced with **S** (Figure 1). Because a significant decrease was observed at 48 hr, this time-point was chosen for subsequent experiments.

### PNA molecules do not interfere with P-gp flow cytometry or P-gp Western blotting assays

**P** and **S** were tested for potential non-specific effects on the efflux assay by adding the same quantity of naked PNA used in a packaging reaction directly to untransduced cells and then performing the assay as described. Analysis confirmed that these PNAs do not interfere with the measurement of efflux capacity (data not shown). The PNAs were also tested for their effect on Western blotting assays. Analysis of the intensities of the bands by ImageJ demonstrated that there was less than 1% difference when PNA was added to cell lysate directly before loading, indicating that Western blotting is an acceptable method for analyzing PNA-mediated reduction of total P-gp expression (data not shown).

### P-gp expression and function are reduced in cells transduced with antigene PNA

**Figure 2:**
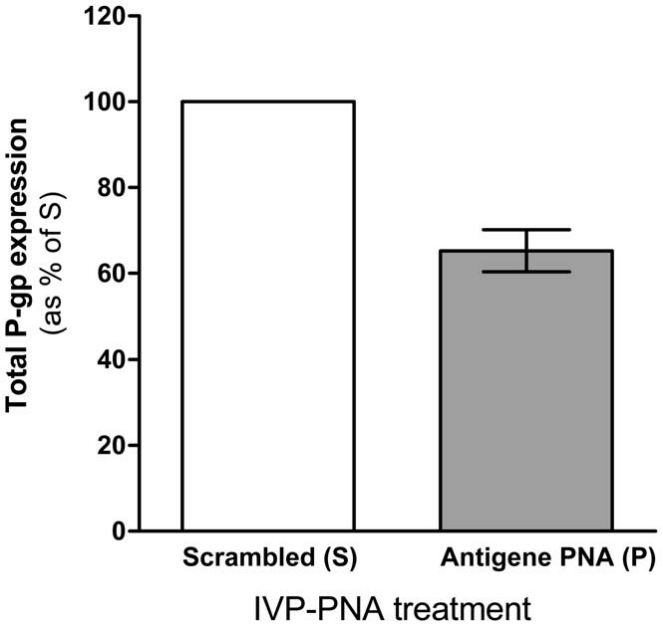
Total P-gp expression in KB-8-5 cells transduced with IVP-scrambled (S) or IVP-antigene PNA (P) was measured by Western blotting using the P-gp specific monoclonal antibody C219; β-actin served as a loading control. The graph represents an average of 4 independent experiments; error bars represent the standard error of the mean. Quantification of P-gp band intensities was performed using ImageJ.

**Figure 3:**
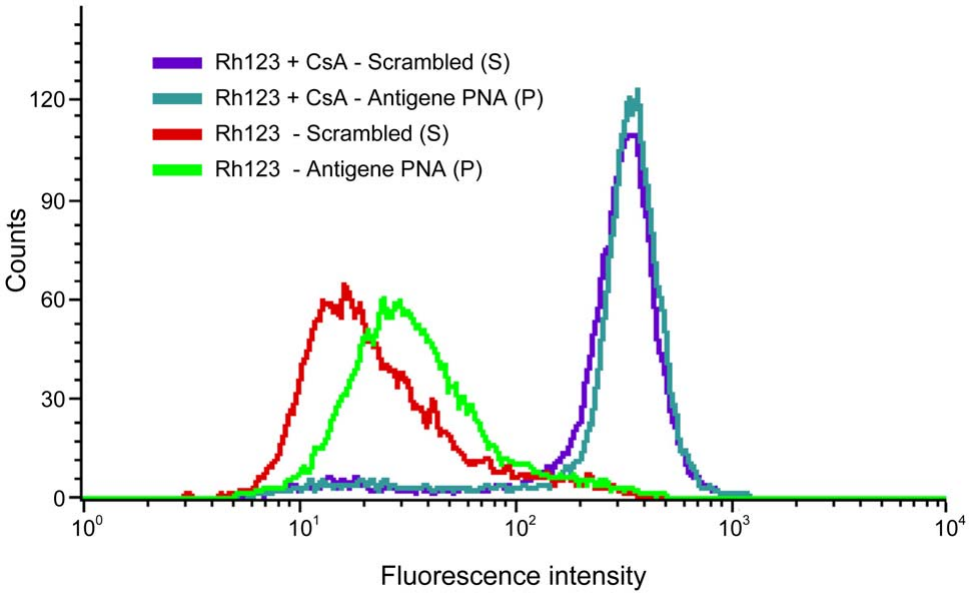
KB-8-5 cells were transduced with IVP-scrambled (S) or IVP-antigene PNA (P) and the P-gp-mediated efflux capacity was measured through the efflux of the fluorescent dye Rhodamine 123 (Rh123), in the presence or absence of a P-gp inhibitor, cyclosporin A (CsA). The coincident histograms of cells transduced with **S** or **P** and treated with CsA demonstrate effective P-gp inhibition. In the absence of inhibitor, cells transduced with **P** show a significant decrease in efflux capacity, evidenced by greater intracellular fluorescence, as compared to cells transduced with **S**.

We sought to test whether the decreased *MDR*1 mRNA levels affected P-gp expression and its efflux function. To that end, KB-8-5 cells were transduced with **P** and **S** via the SV40 delivery system and harvested 48 hr post-transduction. A 35% reduction in total P-gp levels (p<0.01) by Western blotting was observed for cells transduced with **P** as compared to cells transduced with **S** (Figure 2). This result is the average of four independent experiments. The function of P-gp was measured by efflux of Rhodamine 123 48 hr post-transduction using flow cytometry. An accumulation of Rhodamine 123 results in higher intracellular fluorescence, indicating a reduction in cellular efflux capacity. Relative to cells transduced with **S**, cells transduced with **P** via the SV40 delivery system exhibited 38% greater fluorescence (Figure 3).

### Transducing cells twice using IVP-antigene PNA (P) does not substantially reduce total expression or function of P-gp

On the first day of the experiment (Day 1), KB-8-5 cells were transduced with **P** via the SV40 delivery system. On Day 4, the cells were trypsinized, and 1×10^5^ cells were re-transduced. These cells were then analyzed on Day 6 by flow-cytometry and Western blotting as described in Materials and Methods. As a control, KB-8-5 cells transduced with **P** only during the first transduction on Day 1 were also analyzed. P-gp expression was further reduced by 15% (p<0.05) while efflux capacity was reduced by 13% (p<0.05) (data not shown) in cells transduced twice versus once. Although statistically significant, transducing twice is only a slight improvement over a single transduction.

### KB-8-5 cells are more susceptible to paclitaxel when transduced with antigene PNA

**Figure 4:**
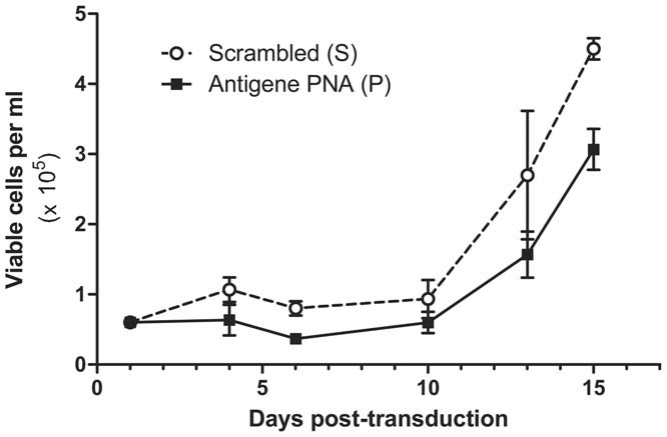
KB-8-5 cells were transduced with IVP-scrambled (S) or IVP-antigene PNA (P) and media containing the chemotherapeutic paclitaxel (400 ng/ml) was added one day post-transduction. Cells were trypsinized every 2–3 days, resuspended in a set volume of media, and counted with Trypan blue. The concentration of viable cells is plotted as a function of time. Error bars represent the standard error of the mean of three replicate counts.

In order to assess the resistance of P-gp-expressing cells to chemotherapy drugs after transduction with antigene PNA, KB-8-5 cells were transduced with **P** and **S** as described in Materials and Methods. One day post-transduction, the medium was replaced by growth media containing paclitaxel (400 ng/ml), a known P-gp substrate. The concentration of viable cells was calculated every 2–3 days (Figure 4). By day 15, a 32% reduction in the number of viable cells was observed for cells transduced with **P** relative to those transduced with **S**. The experiment was repeated twice, independently, with similar results. As a control, cells transduced with **S** were also grown in growth media without paclitaxel.

### Efficiency of the SV40 delivery system

The greatest obstacle in the development of antisense or antigene PNA for therapeutic use is efficient delivery into cells. The *in vitro* delivery of PNA accomplished by Corey and colleagues [23] used a liposome-based system that packaged DNA/PNA complexes. Although protein expression was inhibited using 200 nM concentrations of DNA/PNA, multiple transductions were used. Another strategy that has been employed to deliver PNAs into cells is to use antisense or antigene PNA-peptide conjugates, which have several positively charged amino acids linked to the N-terminus of the PNA [22], [28], [37], [38]. These conjugates were shown to effectively silence a protein product, although PNA concentrations between 1–10 µM were needed, similar to the concentrations used in this report. Another strategy reported in the literature to boost the inhibitory effect of PNA-peptide conjugates was to add calcium ions [30] or chloroquine [38], [39], which aids in endosome disruption, to the media. These agents improved the potency of PNA-peptide conjugates but also increased cell death [28].

One of the most significant advantages of the SV40 delivery system used in our studies is the high efficiency with which these pseudovirion particles deliver constructs to a broad range of cells [40], [41]. Our studies demonstrate that *MDR*1 can be silenced at the transcriptional level *in vitro* by antigene PNA delivered via the SV40 system. We showed that SV40 pseudovirions carrying PNA molecules designed to bind in the region of the major transcription initiation site of the *MDR*1 gene [36] could effectively decrease *MDR*1 mRNA levels and decrease P-gp-mediated efflux capacity in drug-resistant KB-8-5 cells. Significantly, antigene PNA delivered via the SV40 system increased the sensitivity of KB-8-5 cells to a chemotherapeutic agent, a requirement for an effective treatment for P-gp-mediated drug resistance.

In two previous studies [42], [43], antisense PNA was used to knockdown P-gp expression *in vitro*, with results comparable to those presented here. The PNA used in both antisense studies was designed to be complementary to a sequence encompassing the translation start site (−9 to +6) of the *MDR*1 mRNA, and the PNA was either added directly to the cell culture media, or hybridized to DNA and delivered using a lipid-based transfection reagent. Similar to the findings presented here, treating cells with micromolar concentrations of antisense PNA yielded almost a 30% reduction in *MDR*1 mRNA levels and increased the cells' sensitivity to the chemotherapeutic agent adriamycin. In contrast to those studies, our antigene PNA delivered using the SV40 system required much shorter exposures to PNA; transduction times in our study were 2.5 hr, compared to 48 or 96 hr in the antisense studies. Although in the experiments presented here the plates were not aspirated after transduction to remove excess packaged PNA still in the media, our experience has shown that replacing the media after transduction does not significantly alter transduction efficiency. Also, we found that transducing cells multiple times did not significantly increase the silencing effect of the PNA molecules while previous studies using the SV40 system to deliver DNA [44], [45] showed that there was an advantage to multiple transductions.

## Materials and Methods

### Cell culture

The P-gp-expressing, drug-resistant human KB carcinoma cell line KB-8-5 [46] was grown as a monolayer culture at 37°C in 5% CO_2_ using growth media comprised of Dulbecco's modified Eagle's medium (DMEM) supplemented with 10% FBS, 1.8 mM L-glutamine, 90 units/ml penicillin, 90 µg/ml streptomycin, and 10 ng/ml colchicine.

### Synthesis of PNA molecules

PNA was synthesized using standard Boc chemistry as described previously [47], [48]. Methyl benzhydryl amine (MBHA) resin (1.0 g, 0.3 mmol active sites/gram) was downloaded to 0.1 mmol/g with Boc-Lys-(2-Cl-Z)-OH using HATU and DIEA. Any remaining active sites were then capped with a solution of Ac_2_O∶NMP∶pyridine. Downloaded resin (50 mg) was deprotected with 5% m-cresol in TFA, followed by PNA coupling using a 0.4 M solution of PNA monomer in NMP pre-mixed with 0.8 M MDCHA in pyridine and 0.2 M HBTU in DMF. Following coupling, the resin was drained under vacuum and washed and free sites were capped with a mixture of Ac_2_O∶ NMP∶ pyridine. This cycle was then repeated iteratively until the oligomer was complete on the resin. Cleavage from the resin is accomplished under acidic conditions at 0°C using thioanisole, m-cresol, TFMSA, and TFA. Crude product is obtained by repeated precipitation with diethyl ether to obtain white precipitates. The final product is purified by reverse phase HPLC.

### Preparation of in vitro-packaged (IVP) PNA vectors and creation of pseudovirions

Packaging of vectors in this study was performed using scaled-up production methods as previously described [44]. One packaging reaction consisted of VP1-containing nuclear extract from Sf9 cells (100 µg, see Methods S1), which was incubated along with either the *MDR*1-targeted antigene PNA (**P**) or the scrambled control (**S**) (see Table 1) in the presence of 5 mM ATP and 8 mM MgCl_2_ at 37°C for 6 hr in a 600 µL reaction volume to form pseudoviral particles. The PNA concentration during packaging was 25.2 µM. Following incubation, 10 mM CaCl_2_ was added to the reaction tube (0.9 mM final concentration), which was then incubated on ice for 1 hr. *In vitro*-packaged PNA (IVP-PNA) was stored at −20°C.

### Transduction of KB-8-5 cells with IVP-PNA

KB-8-5 cells (1×10^5^) were plated in 60 mm dishes 24 hr prior to transduction in 4 ml of DMEM supplemented with 10% FBS and 1.8 mM L-glutamine (transduction media). On the day of the transduction, the medium was aspirated from the dishes and replaced with 750 µl of fresh transduction media along with 660 µl (one full reaction) of the IVP-PNA. The final concentration of IVP-PNA during the transduction was 10.7 µM. Cells were placed on a rotary shaker at 30 rpm for 2.5 hr at 37°C in 5% CO_2_. Four ml of transduction media was added post-transduction, decreasing the effective PNA concentration more than 5-fold, and the plates were returned to the incubator until analyzed.

### RNA analysis of transduced KB-8-5 cells to measure P-gp mRNA expression

RNA was isolated from KB-8-5 cells 24 hr and 48 hr post-transduction using the Qiagen RNeasy Kit per the manufacturer's protocol with a 15-minute on-column DNAse incubation step. The concentration of RNA was measured with a Nanodrop ND-1000 Spectrophotometer. For each experiment, duplicate measurements of the *MDR*1 mRNA level were made by real-time quantitative RT-PCR using a TaqMan Gene Expression Assay (Assay ID Hs00184491_m1, Applied Biosystems). *MDR*1 mRNA levels were normalized to *GAPDH* (Assay ID Hs99999905_m1, Applied Biosystems).

### Measurement of P-gp-mediated efflux capacity by flow cytometry

KB-8-5 cells were transduced with IVP-PNA as described above and harvested 48 hr post transduction. To assess the efflux capacity of cells transduced with IVP-PNA, cells were incubated with the fluorescent compound Rhodamine 123 (0.5 µg/ml, Sigma) in the presence or absence of 10 µM cyclosporin A (Sigma), a known P-gp inhibitor. Intracelluar fluorescence was then analyzed with a FACScalibur™ (BD Biosciences) flow cytometer running CellQuest™ software (BD Biosciences). Details about incubation times and washing conditions are provided in Methods S1.

### Analysis of total P-gp using SDS-PAGE Western blot

KB-8-5 cells were transduced as described above and lysed 48 hr post-transduction. Proteins in the whole-cell lysates were separated by denaturing SDS-PAGE and P-gp was identified by Western blotting with the primary antibody C219 (Fujirebio Diagnostic). Band intensities were quantified using ImageJ (National Institutes of Health). Specific conditions used for lysis and Western band detection are provided in Methods S1.

### Viability assay

KB-8-5 cells were plated in transduction media and transduced as described above. One day post-transduction, transduction media was replaced with growth media containing 400 ng/ml paclitaxel (Sigma) and the cells were transferred to a T-75 flask (Corning). Every 2–3 days the cells were trypsinized and resuspended in a set volume of media. Live cells were counted using Trypan Blue stain (0.4%; Invitrogen), and then replated into a T-75 flask. The assay was stopped after 15 days.

## Supporting Information

Methods S1 **Supplemental materials and methods.**
(DOC)
