# Supplementary material for: Inhibition of Multidrug Resistance by SV40 Pseudovirion Delivery of an Antigene Peptide Nucleic Acid (PNA) in Cultured Cells
Source: PLoS One. 2011 Mar 22;6(3):e17981. doi: 10.1371/journal.pone.0017981 (PMC3062552; doi:10.1371/journal.pone.0017981)
Supplement: Methods S1 — Supplemental materials and methods. (DOC) [file pone.0017981.s001.doc]

**Inhibition of Multidrug Resistance by SV40 Pseudovirion Delivery of an Antigene Peptide Nucleic Acid (PNA) in Cultured Cells**

Benjamin Macadangdang, Ning Zhang, Paul E. Lund, Andrew H. Marple, Mitsunori Okabe, Michael M. Gottesman, Daniel H. Appella, and Chava Kimchi-Sarfaty

**Supplemental Materials and Methods**

Transduction of Sf9 cells with VP1 baculovirus

A baculovirus construct containing the coding sequence for VP1, the main capsid protein of SV40, was added to 250 ml Sf9 cells with mixing at a multiplicity of infection (MOI) of 10. These cells were harvested 4–5 days later followed by the immediate preparation of their nuclear extract.

Preparation of nuclear extracts from Sf9 cells

Nuclear extracts were prepared as described previously . Briefly, cells were lysed in a buffer containing 10 mM Hepes pH 7.9 (Cellgro), 10 mM KCl, 0.1 mM EDTA, 0.1 mM EGTA (all from Digene), 0.5 mM phenylmethylsulfonylfluoride (PMSF, Fluka), 1 mM dithiothreitol (DTT, Sigma) and Complete™ protease inhibitor cocktail tablet (Roche). 10% Nonidet^TM^-P-40 (NP40) (Sigma) was added and the then nuclei were isolated by centrifugation. Proteins were then extracted from the nuclei by resuspending the nuclear pellet in 20 mM Hepes pH 7.9, 0.4 M NaCl, 1 mM EDTA, 1 mM EGTA, 1 mM PMSF, 1 mM DTT, and a Complete™ protease inhibitor cocktail tablet and vigorously shaking for 15 minutes at 4°C. The extracts were then cleared by centrifugation at 13,000 RPM in a table-top mini-centrifuge for 5 min at 4°C, and the supernatants were stored. DTT, PMSF, and the protease inhibitor cocktail were added to the buffers immediately before use. The protein concentration of the nuclear extracts was measured using the Shaffner-Weissmann Filter Assay . The nuclear extracts were kept for more than a year at -20°C.

RNA analysis of transduced KB-8-5 cells to measure P-gp mRNA expression

Specific oligonucleotide primers and TaqMan probes for *MDR*1 (reference sequence; NM_000927.3) and *GAPDH* were obtained from Applied Biosystems (TaqMan Gene Expression Assay IDs of *ABCB1* and *GAPDH* are Hs00184491_m1, [Hs99999905_m1](https://products.appliedbiosystems.com:443/ab/en/US/adirect/ab?cmd=ABAssayDetailDisplay&assayID=Hs99999905_m1&Fs=y&SearchRequest.Common.PageNumber=1&assayType=ge&chkBatchQueryText=false&SearchRequest.Common.QueryText=GAPDH&srchType=keyword&catID=601267&kwfilter=all&), respectively. For details of TaqMan Gene Expression Assay, see http://www.appliedbiosystems.com/). Synthesis of cDNA from total RNA samples was carried out using TaqMan Reverse Transcription Reagents (Applied Biosystems) with 1 µg total RNA/50 µL reaction volume. The cDNA (1.0 µL RT sample) was amplified using TaqMan Gene Expression Assays (1×) and TaqMan Universal PCR Master Mix Reagents (1×) (Applied Biosystems) in a total volume of 10 µL. The PCR mixture was pre-incubated at 50°C for 2 min, incubated at 95°C for 10 min, and amplified by 40 cycles at 95°C for 15 s and 60°C for 1 min. No-template (water) reaction mixtures were prepared as negative controls. During the PCR amplification, fluorescence emission was measured and recorded in real time on a Roche LightCycler® instrument. Crossing point values were calculated using the ABI PRISM 7900HT software package. The raw results were expressed as number of cycles to reach the crossing point. Crossing point values for *MDR*1 were normalized to the respective crossing point values for the reference gene *GAPDH*.

Measurement of P-gp-mediated efflux capacity by flow cytometry

Transduced cells were collected through trypsinization and washed with PBS (Invitrogen) with 0.1% BSA (Sigma) (PBS/BSA). The function of P-gp was measured by efflux of a fluorescent dye Rhodamine 123 (Rh123) (Sigma). Cells were incubated with 0.5 μg/mL Rh123 in PBS/BSA with or without the inhibitor cyclosporin A (10 μM in PBS/BSA) at 37°C for 10 min (accumulation step). Cells were then washed with PBS/BSA and pelleted by at 485 ×*g*. Cells that were originally incubated with only Rh123 were then resuspended in PBS/BSA and incubated at 37°C for 40 min (efflux step). Cells that were originally incubated with Rh123 and cyclosporin A were then resuspended in 10 μM cyclosporin A in PBS/BSA and incubated at 37°C for 40 min. After the efflux step, cells were washed again with PBS/BSA and analyzed on a FACScalibur™ flow cytometer using CellQuest™ software (BD Biosciences).

Statistical analysis of flow cytometry data

For each PNA treatment, 10,000 cells were counted for each of 3 replicate samples. Statistical analysis was performed using the median fluorescence intensity of each sample. Statistical significance was calculated using a two-tailed t-test.

Analysis of total P-gp using SDS-PAGE Western blot

KB-8-5 cells were plated and transduced as described above. The cells (1 × 10^6^) were collected by trypsinization and centrifugation in a table-top mini-centrifuge at 13,000 RPM for 3 mins. The pellet was resuspended in 100 μL TD buffer (10 mM Tris-HCl, pH 8.0 (Mediatech Inc), 0.1% Triton X-100 (v/v) (RPI), 10 mM MgSO_4_ (Quality Biological), 2 mM CaCl_2_ (Quality Biological)) supplemented with 1% (v/v) Aprotinin (Sigma), 1 mM AEBSF (Sigma), 2 mM DTT (RPI), and 20 μg/mL DNase (Invitrogen). The supplements were added immediately before use. The cell lysates were placed on dry ice for 15 mins and then stored in a -80°C freezer until needed. The cell lysates were thawed in cold water, sonicated three times for 1 minute in a bath sonicator with 30 sec incubation on ice between sonications. Twenty-five μL of SDS-PAGE sample buffer was added to the lysates and incubated at room temperature for 30 min.

After incubation, the samples were loaded on a 7% Tris-Acetate gel (Invitrogen) and run for 90-120 min at a constant voltage of 130 V. The gel was blotted onto a nitroceullulose membrane (Invitrogen) for 1 hour at a constant current of 400 mA. The membrane was blocked using 20% milk in Tris-Buffered Saline with 0.1% Tween-20 (TBST), followed by overnight incubation with the monoclonal antibody C219 (1.5 μg/mL) using 5% milk in TBST, at 4°C. The membrane was then washed three times with TBST for a total of 15 mins and incubated with the secondary antibody Anti-Mouse IgG HRP (RDI) for 2 hr at room temperature (0.4 μg/mL). After washing three times again, Amersham ECL™ Western Blotting Detection Reagents (GE Healthcare) were used as per manufacturer’s instructions and the membrane was exposed on film (Kodak).

**References**

1. Sandalon Z, Dalyot-Herman N, Oppenheim AB, Oppenheim A (1997) *In vitro* assembly of SV40 virions and pseudovirions: vector development for gene therapy. Hum Gene Ther 8: 843-849.

2. Sandalon Z, Oppenheim A (1997) Self-assembly and protein-protein interactions between the SV40 capsid proteins produced in insect cells. Virology 237: 414-421.

3. Schaffner W, Weissmann C (1973) A rapid, sensitive, and specific method for the determination of protein in dilute solution. Anal Biochem 56: 502-514.
